# Adapt, Recycle, and Move on: Proteostasis and Trafficking Mechanisms in Melanoma

**DOI:** 10.3389/fonc.2016.00240

**Published:** 2016-11-15

**Authors:** Seyma Demirsoy, Shaun Martin, Hannelore Maes, Patrizia Agostinis

**Affiliations:** ^1^Laboratory for Cell Death Research and Therapy, Department of Cellular and Molecular Medicine, KU Leuven, Leuven, Belgium; ^2^Laboratory for Cellular Transport Systems, Department of Cellular and Molecular Medicine, KU Leuven, Leuven, Belgium

**Keywords:** melanoma, proteostasis, autophagy, unfolded protein response, exosomes

## Abstract

Melanoma has emerged as a paradigm of a highly aggressive and plastic cancer, capable to co-opt the tumor stroma in order to adapt to the hostile microenvironment, suppress immunosurveillance mechanisms, and disseminate. In particular, oncogene- and aneuploidy-driven dysregulations of proteostasis in melanoma cells impose a rewiring of central proteostatic processes, such as the heat shock and unfolded protein responses, autophagy, and the endo-lysosomal system, to avoid proteotoxicity. Research over the past decade has indicated that alterations in key nodes of these proteostasis pathways act in conjunction with crucial oncogenic drivers to increase intrinsic adaptations of melanoma cells against proteotoxic stress, modulate the high metabolic demand of these cancer cells and the interface with other stromal cells, through the heightened release of soluble factors or exosomes. Here, we overview and discuss how key proteostasis pathways and vesicular trafficking mechanisms are turned into vital conduits of melanoma progression, by supporting cancer cell’s adaptation to the microenvironment, limiting or modulating the ability to respond to therapy and fueling melanoma dissemination.

## Melanoma

Cutaneous malignant melanoma (or simply melanoma) is a skin cancer that arises from the malignant transformation of melanocytes, the essential pigment (melanin)-secreting cells of the skin, whose incidence is quickly rising worldwide, and in particular in the developed countries (1). In the last decade, melanoma has emerged as a paradigm of aggressive cancer, typified by a high heterogeneity and poor therapeutic response. In fact, unless diagnosed at an early stage and surgically resected, melanoma evolves rapidly as a metastatic disease, highly resistant to therapy and associated with poor prognosis. Moreover, despite efforts for early detection and prevention, the median survival of patients with metastatic melanoma remains dismal (6–12 months) (2).

Crucial melanoma-associated oncogenic events have been identified, including a large variety of genetic and epigenetic defects in key components of the cell cycle, cell death, and epithelial-to-mesenchymal-like transition (EMT). Distinctive mechanisms related to the aggressive behavior of melanoma cells are the constitutive activation of growth factor receptors, such as c-Kit, PDGFR-α, EGFR, and the RAS/RAF/MEK/ERK pathway (or just MAPK pathway) (3, 4). It is known that 40–60% of all melanoma patients harbor a somatic mutation in the 600 residues of BRAF, an early event in melanomagenesis, with 80% of these patients displaying V600E mutations, while 20% and a very small population of approximately 5–7% of patients harboring V600K or V600R mutations, respectively (5, 6). Moreover 20, 2, and 1% of all melanomas are due to mutations in NRAS, KRAS, and HRAS, respectively, with the most common NRAS mutation occurring at position Q61 (7). Other known oncogenic mechanisms relate to the heightened activation of the PI3K/protein kinase B (AKT) pathway (partially driven by the loss, mutation, or epigenetic silencing of the PTEN tumor suppressor) (8–10), transcription factor NFκB, overexpression of the metabotropic glutamate receptor 1 (GRM1) (11), and dysregulation (deletions, silencing, mutations) of genes involved in the cell cycle (CDKN2A/CDK4/CCND1) or regulating apoptosis, such as MDM2/4 and Apaf-1, and anti-apoptotic Bcl-2 family members, such as Bcl-x_L_ and Mcl-1 [extensively reviewed in Ref. (12–15)]. Moreover, p53 has been recently shown to be a *bona fide* ultraviolet radiation (UVR) target gene in melanoma, and acquired, UVR-induced p53 mutations accelerate BRAFV600E-driven melanomagenesis (16).

In addition to driving unrestrained proliferation and increasing the resistance to apoptosis, these genetic and additional epigenetic alterations also modulate the cell autonomous ability of melanoma cells to invade and migrate (e.g., through altered expression of adhesion proteins). Moreover, both heightened NFκB signaling (17) and increased GRM1 expression (18), which foster glutamate-mediated MAPK-driven melanoma cell survival and the AKT–mTOR–HIF1 pathway (19), have been shown to support melanoma-associated proangiogenic signaling, thereby favoring melanoma growth and dissemination. Finally, the complex gene expression landscape of melanoma is further regulated by several epigenetic mechanisms, including methylation, chromatin modification and remodeling, and through various classes of non-coding RNAs (20, 21).

Although melanoma can be considered a prototypical immunogenic tumor, it is a very aggressive cancer that can progress even in the presence of significant lymphoid infiltrate or demonstrated antitumor immune responses (22). This is largely due to the ability of melanoma to efficiently escape the immune system through various mechanisms [e.g., by the release of immune-silencing molecules such as vascular endothelial growth factor (VEGF), transforming growth factor (TGF)-β, interleukin (IL)-10, nitric oxide (NO), or prostaglandins], in part relying on the enhanced secretory activity of melanoma cells. Together, these observations suggest that deranged melanoma cell autonomous processes and melanoma cell–stromal cell interactions contribute to the establishment of a tumor-promoting and immunosuppressive microenvironment driving melanoma growth.

Based on the accumulating knowledge on melanoma’s malignant features, in recent years, different classes of novel drugs were approved for the treatment of advanced melanoma. However, targeted molecular therapies relying on blockage of altered MAPK pathway, such as BRAFV600E (e.g., vemurafenib) and MAPK/ERK kinase (MEK) (e.g., trametinib) inhibitors (23), have demonstrated only partial antitumor responses in patients (24, 25). This is largely due to the rapid emergence of drug-resistant (6) and more aggressive melanoma clones (26), reflecting the high degree of cellular plasticity of melanoma cells and possibly pre-existing heterogeneity, which is one of the major hallmarks of this aggressive disease. An important emerging class of anti-melanoma drugs targets co-inhibitory receptors limiting T cell-mediated antitumor responses through the agency of anti-CTLA-4 or anti-PD-1 or PD-L1 antibodies (27). However, despite the very promising objective clinical responses, it has become clear that a sizeable subset of melanoma patients does not respond equally well to immune checkpoint blockade strategies, which are often accompanied with severe toxic side effects (28). Thus, the limits and undesired side effects of current anti-melanoma therapeutics create a great interest to investigate new targets and develop novel approaches.

Recent data underscore that during melanoma progression certain housekeeping processes are used by melanoma cells to adapt and to sustain oncogene- and aneuploidy-driven dysregulations, much more than normal melanocytes would need them. In keeping with this, accumulating evidence indicate that, to support their intrinsic plasticity, melanoma cells rewind key homeostatic pathways, such as UPR, vesicle trafficking, and key lysosomal pathways, like autophagy. These pathways have been shown to be dynamically regulated throughout melanoma progression to increase intrinsic adaptations against proteotoxic stress, to accommodate the high metabolic demands of these cancer cells, and to modulate the interface with stromal cells within the tumor microenvironment. However, unlike other well-documented types of stress involved in malignant behavior, such as genotoxic, oxidative, and metabolic stress in cancer cells, much less is known about the mechanisms regulating proteostatic stress and how perturbations in the proteome of cancer cells, and more specifically in melanoma, affect disease progression, despite its prominent manifestation in other human disorders (29).

In this review, after a brief introduction of the main pathways regulating proteostasis, we discuss in more molecular details altered mechanisms governing the proteome of melanoma cells and how they modulate melanoma survival, metastatic spreading, and therapy responses.

## The Proteome Under Control: A Brief Overview of Key Pathways Regulating Proteostasis

**Figure 1:**
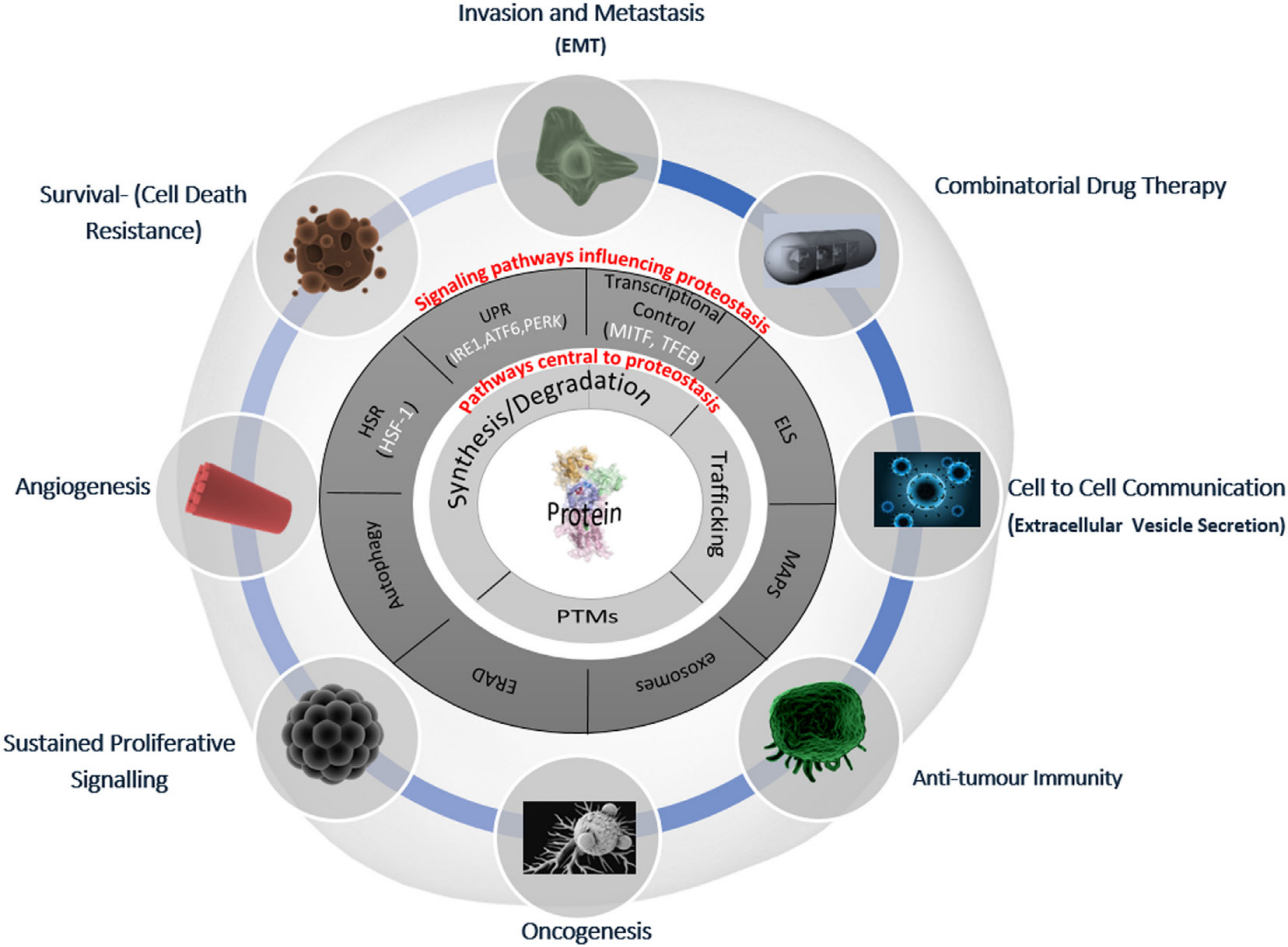
**Deranged proteostasis network in melanoma supports the main hallmarks of cancer**. A simplified overview of the main components of the proteostasis network is discussed in this review and its implications for melanoma biology. The deranged proteostasis network (PN) in the depicted melanoma cell; the inner-most circle (light gray) represents the general proteostasis control (GPC) triad (35) and includes the synthesis, degradation, posttranslational modification, and trafficking of proteins. The second circle (dark gray) shows the signaling pathways that influence the level and activity of the triad. The outer radial circle gives the summary of the key melanoma-associated processes, which have been shown to be affected/modified by alterations of the PN [adapted from Ref. (36)]. EMT, epithelial mesenchymal-like transition; HSR, heat shock response; UPR, unfolded protein response; ELS, endo-lysosomal system; ERAD, ER-associated degradation; MAPS, misfolded-associated protein secretion; PTMs, posttranslational modifications.

The regulation of intracellular protein turnover (synthesis/degradation), protein conformation/folding, protein–protein interactions, and localization (trafficking) are crucial processes preserving the quality of the proteome (proteostasis), which is required for a cell to function efficiently and to dynamically respond to internal and external cues. In line with their role in the maintenance of a healthy proteome, various proteostasis mechanisms have been shown to be disturbed during aging as well as in many pathological conditions, including cancer and neurodegeneration (30, 31). Proteostasis is fine-tuned by vital protein quality control mechanisms, including autophagy and the proteasomal and endo/lysosomal pathways, which make up the triad of modalities that encompass general proteostasis control (GPC, Figure 1). These modalities utilize various classes of chaperones, folding enzymes, posttranslational modifications, synthesis, trafficking and degradation mechanisms, which work in concert and are modulated by stress pathways, ultimately sensing changes in the integrity of the proteome. In the following paragraphs, we will provide a simplified introduction on the main mechanisms contributing to proteostasis; a more detailed discussion is beyond the scope of this essay and can be found in excellent recent reviews (32–34).

To maintain proteostasis and prevent/minimize proteotoxic stress as a result of the accumulation of aged, aberrantly folded, or aggregated proteins, cells are heavily dependent on various quality control mechanisms. Cytoplasmic proteostasis is vitally regulated by *the heat shock response (HSR)*, which is largely mediated by the stress-induced activation of heat shock transcription factor 1 (HSF1). Upon cellular stress, the activation of HSF1, the master regulator of the HSR, causes the induction of the expression of HSPs, molecular chaperones that protect the proteome against misfolding and subsequent aggregation, by facilitating folding, transport, and degradation (32). HSPs exert their first line-quality control activity by assisting folding of *de novo* synthesized protein during their transit within cellular compartments, facilitating refolding of stress-denatured proteins, oligomeric assembly, protein transport, and providing assistance in proteolytic degradation. Moreover, HSPs assist in the prevention of protein aggregation by shielding the exposed hydrophobic surfaces of partially unfolded proteins (34).

While HSR regulates cytoplasmic proteostasis, *the unfolded protein response (UPR)* is activated in response to changes in ER folding capacity and regulates ER proteostasis.

The transcriptional program that is put in motion by the activation of the UPR preferentially acts as an intermediate response to reestablish the ER protein folding and synthesis output as a consequence of increased detection of unfolded or misfolded proteins in the ER lumen (33) (see Box 1 for molecular description of the UPR). During the initial phase, the UPR is an adaptive response that potentiates a diverse array of quality control mechanisms aiming to reestablish ER folding capacity and homeostasis. This is mainly achieved by temporarily shutting down protein synthesis, to reduce folding burden in the ER lumen, while increasing the expression of various chaperones and folding enzymes (33). Besides the UPR emanating from the ER (UPR_ER_), recent studies have implicated another transcriptional, mitochondrial stress response, called the mitochondrial unfolded protein response or UPR_mt_, as major contributor of proteostasis. Although partially overlapping with elements of the HSR and UPR_ER_ and not entirely understood in mammalian cells, the UPR_mt_ program is elicited to promote folding, limit import, and reduce translation of mitochondrial proteins and is selective for mitochondrial chaperones, such as the mitochondrial HSP60 (37). However, if stress persists and proteostasis is not reestablished, both UPR-signaling mechanisms activate mechanisms capable of inciting cell death. In the scenario of the best characterized UPR_ER_, this involves increased expression of the transcription factors ATF4 and induction of CHOP, downstream in the PERK–eIF2α branch of the ER-stress response (38).

Box 1 **The unfolded protein response.**

Loss of the ER folding capacity leads to the accumulation of unfolded or misfolded proteins and the initiation of the unfolded protein response (UPR). The UPR is sensed by the luminal domain of three ER-stress transmembrane proteins, which are PERK, IRE1 – the latter harboring both protein kinase and endoribonuclease activities –, and ATF6. Binding of GRP78 (also called BiP) to the luminal domain of these ER transmembrane proteins keeps them inactive. Upon loss of ER homeostasis, which causes an accumulation of unfolded proteins, GRP78 is titrated away from these ER-stress sensors to bind to the hydrophobic domains of misfolded proteins, allowing the dimerization/oligomerization of PERK and IRE1α, and the migration of ATF6 to the Golgi, ultimately launching the UPR (33). Active *PERK* attenuates global protein synthesis by the phosphorylation of eIF2α, thus relieving pressure on the stressed ER (39, 40), while at the same time allowing cap-independent translation of mRNA important in both pro-survival and pro-death stress response proteins (41). An essential role here is played by the activating transcription factor 4 (ATF4), which stimulates the synthesis of essential ER chaperones, proteins involved in ER homeostasis (42) as well as autophagy (43). However, ATF4 can also induce the expression of the pro-apoptotic transcription factor C/EBP homologous protein CHOP, enabling the PERK branch of the UPR to elicit cell death under conditions of unresolved ER stress (44). The antioxidant response element (ARE) transcription factor Nrf2 is also induced by PERK and is a homeostatic response to counter reactive oxygen species (ROS) (45). Negative feedback by kinase inhibition of PERK (46) and eIF2α dephosphorylation (47) as stress is resolved, restores the cell’s normal translational capacity. *IRE1* displays protein kinase and endoribonuclease activities. Oligomerization and autophosphorylation of IRE1 results in RNase-domain activation leading to unconventional splicing of the XBP1 mRNA (48). XBP1 splicing generates an active transcription factor that upon nuclear translocation induces the expression of several cytoprotective genes involved in ER quality control, such as ER-resident chaperones involved in protein folding and the tagging of terminally mutated proteins for ER-associated degradation.(ERAD). IRE1 can also degrade specific mRNAs through a process termed regulated IRE1-dependent decay of mRNA (RIDD). As in the case of PERK, while XBP1splicing is mostly involved in evoking cytoprotective responses, IRE1 can engage cell death pathways as well. This can occur either through its scaffolding role at the ER leading to the activation JNK (49) and through RIDD-mediated degradation of pro-survival mRNAs, which increases with the intensity of ER stress (50). Following release from the ER, *ATF6* translocates to the Golgi apparatus where it is cleaved by Golgi-resident proteases into an active transcription factor (51), which binds to promoters containing ER-stress response elements (ESRE) and regulates the expression of key enzymes and chaperones of the UPR machinery (52).

Although PERK, IRE1, and ATF6 each appear to play functionally separate roles in stress signaling, conserved overlap in chaperone induction and cell death activation exist, further highlighting the importance of cellular proteostasis and how it is coupled to cell death pathways.

Both the HSR and UPR-signaling pathways work in conjunction with major proteolytic systems, such as the proteasome, autophagy, and the endo-/lysosomal system (ELS) (33, 53), to maintain the integrity of the proteome and attenuate proteotoxicity, thus establishing a complex network of interconnected proteostasis mechanisms.

Typically, under conditions of loss of ER proteostasis misfolded/abnormally, folded proteins are re-translocated to the cytoplasm and subsequently degraded by the ubiquitin–proteasomal system (UPS) through a process called ERAD [reviewed in Ref. (43)]. Alternatively, ER protein overload can be alleviated by the secretory pathway, through a newly described mechanism whereby unfolded glycosylphosphatidylinositol (GPI)-anchored proteins are exported *via* the Golgi apparatus to the plasma membrane (PM) for subsequent endocytosis and lysosome degradation (54).

In the cytosol, non-functional/damaged or misfolded proteins are sensed and chaperoned. Failure to refold the target proteins, induces the activity of E3 ubiquitin ligases, including the co-chaperone carboxyl terminus of co-chaperone carboxyl terminus of heat-shock cognate 70 (HSC70)-interacting protein (CHIP), resulting in the ubiquitination of unfolded/damaged proteins and their targeting for degradation through the UPS (55). The ubiquitin-tag, is a well-known posttranslational modification allowing polypeptides to be targeted and delivered to the 26S proteasome for degradation (56–59). However, if the capacity of the chaperone-mediated refolding machinery and the UPS are overloaded, or if the misfolded proteins form large aggregates that are resistant to proteasomal degradation, these ubiquitin-modified substrates are recognized and targeted to the lysosome through autophagy-mediated pathways. In line with this, in addition to the ERAD pathway, autophagy has been shown to play an important role in restoring ER proteostasis (60).

Protein ubiquitination has been moreover recognized to be a crucial signal for the selection and delivery of cytoplasmic cargo, not only restricted to aberrantly folded proteins and aggregates but also to damaged organelles (like the mitochondria), to the autophagy machinery. This mechanism confers specificity to the process of autophagy, which was initially thought to occur in an aspecific manner. Ubiquitinated cargo is recognized by the binding of shuttling factors or specific receptors, of which p62 (also called sequestosome 1 or SQSTM1) and neighbor of BRCA1 gene 1 (NBR1) are the prototypes, that tether it to the autophagosomes, the hallmark of macroautophagy (thereafter called simply autophagy, see Box 2 for further description). During *autophagy*, damaged or aberrant cytoplasmic material is sequestered by double-membraned autophagosomes and trafficked to the lysosomes, where upon fusion, is ultimately degraded by lysosomal hydrolytic enzymes. However, autophagosomes can fuse with an associated membrane protein 1 (LAMP1)-positive compartment before acquiring lysosomal proteases and enter the endocytic pathway through fusion with late endosomes (LEs), forming the amphisomes and hybrid organelles (61). Although a complete description of the molecular pathways regulating autophagy is beyond the scope of this review [for further details, readers are referred to Ref. (62–64) and Box 2], it is important to note that the ER plays an instrumental role in the modulation of autophagy and is thought to provide an important membrane source during the early steps of autophagosome formation (65).

Box 2 **Autophagy pathways.**

To date, three different mechanisms have been identified in mammalian cells, which facilitate the lysosomal degradation of intracellular components: chaperone-mediated autophagy (CMA), macroautophagy, and microautophagy.

*Macroautophagy* is the major lysosomal degradation pathway hallmarked by formation of a double-membraned autophagosomes (48, 49). Dysfunctional/obsolete organelles and proteins, both soluble and aggregates that are tagged for recycling, are encapsulated in the forming autophagosomes that ultimately fuse with the lysosome (autolysosomes) for degradation (66). At the molecular level, the induction of autophagy is regulated by two kinase complexes; the ULK1 complex that includes ATG13, FIP200, and ATG101, and is suppressed by mTOR and the large class III PI3P–Vps34 complex, which includes among others Beclin 1, which generates PI3P on the nascent autophagosome. These kinase complexes in turn coordinate the recruitment of downstream autophagic proteins, including WIPI1 and 2, the membrane associated ATG9, two ubiquitin-like protein complexes ATG12–ATG5–ATG16 and the LC3/GABARAP (ATG8) proteins which, upon phospatidylethanolamine (PE)-lipidation, become integrated in the autophagosomal membrane and become degraded upon fusion with the lysosome (62, 67, 68). Selective macroautophagy pathways can lead to the clearance of specific targets, such as protein aggregates (aggrephagy), organelles such as mitochondria (mitophagy), endoplasmic reticulum (reticulophagy), and peroxisomes (pexophagy), or lipid droplets (lipophagy), glycogen particles (glycophagy), and pathogens (xenophagy). In analogy to the proteasome, selective cargo recognition and autophagic degradation also involves ubiquitination. The autophagic degradation of protein aggregates requires the ubiquitin receptors p62/SQSTM1 and NBR1, which recognize polyubiquitinated targets and bridge them to the autophagy machinery. Both autophagic adapters are cargo receptors and autophagy substrates (69) and share similar domain architecture, interacting with LC3/Atg8 through an LC3-interacting region (LIR) and binding to monoubiquitin and polyubiquitin *via* the C terminal ubiquitin-associated (UBA) domain.

*Chaperone-mediated autophagy* involves the selective degradation of soluble proteins exposing a KFERQ-related sequence, which are directly targeted to the lysosomes upon their recognition by the cytosolic heat shock cognate 70 (hsc70) (70). The substrate-chaperone complex interacts with the lysosome-associated membrane protein-2A (LAMP-2A) receptor, which ensures its translocation into the lysosome assisted by lysosomal hsc70. This process encompasses four main steps, which are as follows: (a) substrate recognition and targeting to the lysosomes; (b) substrate binding to the lysosomal receptor and unfolding; (c) substrate translocation through the lysosomal membrane; and (d) substrate degradation in the lysosomal lumen (71).

*Microautophagy* is the third subtype of autophagy, which to date has not yet been well characterized in mammalian cells (72). It involves the internalization of cytosolic cargo through invagination of the lysosomal membrane, through a process that resembles the formation of multivesicular bodies (MVBs) (72). Although the molecular players have not been well defined and the relationship between this process and the formation of MVBs is elusive, microautophagy underlies the ability of the lysosomal membrane to direct engulf cytosolic components. During this phenomenon, a specialized “autophagic tube” is formed by invagination of the lysosomal membrane, which encloses portions of the cytosol through an ATP-dependent process that is accompanied by drastic changes in the distribution of both lysosomal lipids and proteins. In mammalian cells, non-selective microautophagy clears soluble intracellular substrates, whereas selective microautophagy mechanisms have only been described in yeasts.

While these autophagy pathways are constitutively active, macroautophagy and CMA are stimulated in response to a variety of common metabolic and oxidative stressors, and mutual compensatory mechanisms exist to balance and compensate these degradation pathways (70). Microautophagy, is often stimulated in parallel with macroautophagy, especially in response to starvation or mTOR inhibition, and is thought to be a mechanism important to reestablish lysosomal membrane homeostasis and regulate lipid metabolism and endocytosis (73).

Additionally, proteostasis is regulated at different levels through the *ELS* (schematically described in Box 3), which interfaces and overlaps extensively with the autophagy machinery by sharing a number of tethering, fusion, and trafficking components (74).

Box 3 **The endo-lysosomal system.**

The endo-lysosomal system (ELS) is a dynamic, interconnected vesicular network including a set of intracellular membranous compartments, which comprise the endocytic pathway (early endosomes, recycling endosomes, late endosomes, and lysosomes) (75). Endocytosis is initiated by invagination of a portion of the plasma membrane, typically through the action of chlatrin-coated pits, or through plasma membrane caveolae, or through larger vesicles like micropinosomes. Upon internalization, endocytic vesicles fuse with the mildly acidic early endosomes, to be finally transported to the acidic compartments, namely the late endosomes (LEs) and finally the lysosomes for degradation. In the ELS, the maturation of endosomes into LEs/lysosomes entails a significant shift in the acidity of the vesicles, a process that ultimately regulates vesicle trafficking, protein sorting, and targeted degradation of sorted cargo. These vesicular pathways involve various membrane fusion events that are vitally regulated by members of the Rab small GTPase family, tethering complexes, and actual membrane fusion events guided by SNARE proteins (51) and posttranslational modifications, primarily ubiquitinilation. The early endosomes are regulated predominantly by the small GTPase Rab5 (52) and act as the major sorting station holding the capacity to go back to cell surface for reuse (recycled endosomes), a process under the regulation of Rab 4 and 11 (76, 77), or mature into late endosomes (LEs), which is facilitated by the transition from Rab5 to Rab7. The major roles of LEs are the biogenesis of intraluminal vesicles and sorting of ubiquitinated membrane proteins for lysosomal degradation (78). An LE demonstrating intraluminal vesicle (ILVs) formation, in a process of inward membrane invagination involving the ESCRT complexes, is referred to as a multivesicular body (MVBs) (79). In the end, the MVBs/LEs fuse with lysosomes for degradation. However, MVBs can also fuse with the plasma membrane leading to the release of their internal vesicles into the extracellular space (exosomes) (80).

An intense cross talk exists between trafficking mechanisms and molecular modulators of autophagosomes and ELS. For example, autolysosome formation and the trafficking of MVBs to lysosomes are processes regulated by LE-associated proteins, among which the small GTPase, Rab7A, a critical regulator of endosomal maturation/functionality (81–83) and syntaxin 5 (84). Moreover, endosomal membranes can contribute to the formation of autophagosomes through vesicles derived from Rab11-positive recycling endosomes, where both ULK1 and mATG9 reside and sense autophagy induction (85). Additionally, mATG9 and ATG16L1-containing vesicles can traffic through the endocytic pathway *via* different routes and fuse with recycling endosomes (86). These examples clearly show that autophagosomes can undergo fusions with both acidic vesicles of the endo-lysosomal system as well as earlier compartments of the endocytic pathway.

At the level of the LEs/MVBs and lysosomes, proteostasis is performed by a variety of differing mechanisms, chiefly regulated by ubiquitin signals, including, protein sorting (recycling) and targeted clearance (degradation) of the cargo (87). Furthermore, LE/MVBs can fuse with the cell membrane to release proteolytic enzymes and signaling molecules or ILVs, called exosomes, thereby regulating intercellular communication (80).

A number of studies have further highlighted the close cross talk between autophagy and the UPS (88–91). Moreover, perturbed autophagy may also lead to the impaired degradation of specific UPS clients; such that reduced autophagic capacity can alter the efficiency of the proteasome through mechanism regulated by the key autophagic receptor p62 (55, 90, 91). This suggests that the autophagy and UPS proteolytic machineries are functionally linked and do not simply respond to compensatory mechanisms. Moreover, evidence coupling the proteasomal and endosomal machinery is also emerging (92, 93).

Recently, the autophagy machinery (94) and the UPS (94) have been shown to be implicated in unconventional secretion pathways, facilitating the extracellular delivery of cytosolic proteins directly from the cytosol, without transiting trough the classical ER-to-Golgi pathway. In case of the UPS-mediated pathway, it has been suggested that this unconventional secretory mechanism, called misfolded-associated protein secretion or misfolded-associated protein secretion (MAPS) and involving the LE as delivery carriers, may help proteostasis through the export of misfolded proteins when the proteasomal degradation capacity in the cell is overwhelmed, a condition that may lead to the accumulation of detrimental protein aggregates (94).

In conclusion, although the molecular nature of all these interactions and the identity of the shared components have not been fully characterized, it is clear that there is a more significant cross talk within the complex network of the pathways regulating cellular proteostasis and protein quality control, than originally hypothesized. This is perhaps not surprising considering that ubiquitin-regulated pathways, autophagosomes, endosomes, and lysosomes are emergently recognized as highly dynamic and cross-communicating signaling hubs controlling not only intracellular protein/organelle degradation but also the strength and duration of signaling pathways originating from intracellular and surface receptors/complexes as well as the composition of the surface proteome and secretome (95–98).

In the following sections, we will discuss how these major housekeeping pathways are recruited to support different features of melanoma plasticity and aggressiveness.

## Adapt and Survive: Alteration of Cytoplasmic and UPR-Based Proteostasis in Melanoma

Cancer cells are particularly exposed to a variety of intrinsic (oncogenic expression and aneuploidy) and extrinsic perturbations (hypoxia, glucose deprivation, acidosis) that alter global proteostasis, resulting in the accumulation of flawed or misfolded proteins. Moreover, to keep pace with their increased metabolic/energy request and heightened proliferation, cancer cells usually expand their protein folding and trafficking capacities. Indeed, oncogenic expression and chromosome imbalance, characteristic of malignant cells, impose pressure on proteostasis control and encumber cellular quality control mechanisms, mainly due to imbalances in protein synthesis, e.g., excessive/overexpression of proteins that often culminate in protein aggregation and the activation of degradation and/or export pathways. To cope with these cues, cancer cells tend not only to upregulate various mechanisms regulating proteostasis, by e.g., upregulating chaperones and expanding their secretory/vesicular pathways but also become heavily reliant on them – a phenomenon known as “non-oncogene addiction” (99).

In this context, melanoma represents a key paradigm. Accumulating evidence shows that melanoma cells display a significant increase in ER and cytoplasmic chaperones, driven by the UPR (Box 1), which act initially to increase protein folding efficiency, along with an elevated expression of many vesicular trafficking related genes, such as Rab7 (100), TBC1D16 (101), and Rab27 (102). Moreover, the relevant role that chaperones (for example; HSP70, HSP90) play in preserving melanoma survival and plasticity is emphasized by their correlation with disease progression (103, 104) and the manifested vulnerability that melanoma cells exhibit following perturbations of global proteostasis. A study comparing the expression levels of HSPs across patient clinical parameters showed that, with the notable exception of HSP32, whose expression correlated with improved patient survival, increased expression of HSP90 and HSP40 correlated with advanced stages of melanoma, and in the case of HSP40 with decreased patient survival (105). In line with this, another study observed an indispensable role of HSF1 in melanoma progression and migration, thus highlighting its potential as therapeutic target in melanoma (106).

Increased chaperone expression may also allow for heightened protein synthesis, feeding melanomas proliferative capacity (107). Moreover, chaperones are multifaceted proteins that modulate signaling pathways as a consequence of their high affinity for unfolded or misfolded proteins, drawing them away from their regulatory role in signaling pathways. For example, HSP90 is an essential cytoplasmic chaperone, renowned for its protective capacity against a number of chemotherapeutics and implicated as a modulating factor of drug resistance (108). Over 100 client proteins have been identified for HSP90, many of which are important in the progression of cancer (3, 4), including MEK, AKT, Apaf-1, VEGFR, and c-MET (109). Moreover, the chaperone HSP90 stabilizes mutant BRAFV600E, further supporting the prognostic relevance of HSP90 upregulation in melanoma and the close correlation between oncogenic drivers and increased chaperone expression (108).

Interestingly, a recent study showed that upon cellular stress HSF1 physically interacts with and is directly phosphorylated by MEK, a pivotal kinase of the MAPK pathway, and as a consequence promotes melanoma growth (110). Furthermore, MEK inhibition in these BRAFV600E mutant melanoma cells, caused HSF1 inactivation, protein destabilization, and aggregation that lead to melanoma cell demise by excessive proteotoxic stress. Interestingly, MEK–HSF1 inhibition or proteasomal inhibition, also led to the accumulation of aggregation-prone proteins forming amyloid fibrils enriched in beta sheet structure, through the process of amyloidogenesis. Increased amyloidogenesis by MEK or HSF1 inhibition blunted melanoma growth, by exacerbating proteotoxic stress to a lethal threshold, thus suggesting that the induction of proteotoxic stress might have relevant anti-melanoma effects and represent a promising therapeutic strategy (110).

Unfortunately, the use of bortezomib, a clinically used 26S proteasome inhibitor, for the treatment of melanoma gave disappointing results (111). One rationale for this was the tendency of bortezomib to upregulate ER stress, cytoprotective autophagy, and the detectable expression of key cytoprotective chaperones, such as HSP70, all of which are potential therapeutic targets in melanoma (112). In line with this, targeting HSP70 significantly increased the efficacy of bortezomib to incite cell death and reduced the metastatic potential of melanoma (112). In agreement with this strategy, while single agent MEK inhibition or bortezomib were shown to be only partially beneficial, their combination markedly synergized their antitumor and anti-metastatic potential in a xenografted model of melanoma (110).

Taken together, from a cell focused perspective, these observations suggest that targeting multiple nodes of the proteostasis network of melanoma cells would significantly enhance melanoma cell’s vulnerability to proteotoxic stress.

Melanoma cells are moreover renowned for their chronic activation of the UPR_ER_ (107), and a significant proportion of research has committed to understanding the molecular and therapeutic potential of this adaptation [reviewed in Ref. (107)]. The importance of ER homeostasis in melanoma is highlighted by the constitutive upregulation of certain ER-resident chaperones, such as GRP78, and their correlation with upstream, disease initiating, NRAS, or BRAF oncogenic mutations (107). In fact, ample evidence indicates GRP78 as a prognostic marker of melanoma, where its increased expression correlates with disease progression (113). The observed enhancement in basal UPR_ER_ activation in melanoma cells is in part justified by the increased protein synthesis burden required for proliferation, driven by the constitutively activation of the MAPK pathway and AKT, downstream mediators of the RAS–RAF and PI3K pathways, respectively (107). However, while some studies have shown that inhibition of BRAF or MEK signaling in melanoma increases ER stress, yet conversely, others have demonstrated that inhibition of oncogenic BRAF or MEK resulted in a marked reduction in IRE1 and ATF6 based signaling (107) (Box 1). Interestingly however, the capacity of the RAS–RAF pathway to modulate PERK-based signaling was not altered (107).

Various HSP proteins, including HSP70, are also found at the cell surface and/or are secreted from melanoma cells under stress conditions eliciting loss of proteostasis. Increased expression of HSP70 in melanoma cells triggered its rerouting through the ELS, allowing excess HSP70 to be deposited to the cell surface and be released into the extracellular space (103). This suggests that stress conditions altering HSP expression in melanoma cells may concomitantly favor their extracellular release through the ELS.

However, the extracellular route of delivery, and especially the context in which chaperones are exposed or released, whether they originate from living or dying cells, modulate their known extracellular immunomodulatory activities. During immunogenic cancer cell death (ICD), a cancer cell death subroutine triggered by a limited set of assorted anticancer therapies resulting in the efficient stimulation antitumor immunity (114), vital homeostasis processes favor the trafficking and export of key chaperones to the surface of the dying cells, where they act as damage-associated molecular patterns (DAMPs). During ICD, loss of ER proteostasis and an intact secretory pathway were found to be required for the surface exposure of the ER luminal chaperone calreticulin (CRT), a major ICD-associated danger signal, whose exodus is often accompanied by the extracellular exposure or release of HSP70 (115). Notably, in melanoma cells dying in response to the genotoxic agent melphalan (Mel), the limited induction of an ER-stress response failed to elicit the surface emission of CRT and HSP70 (116) and to efficiently stimulate a CD8^+^ T cell-dependent antitumor response, in a prophylactic immunization mouse model (116). Intriguingly, the anticancer vaccination potential of Mel was potentiated by the exogenous addition of recombinant CRT (116). This concept is further highlighted by the observation that upon ROS-induced loss of ER proteostasis, autophagy counterbalances the mobilization of CRT on the surface of dying melanoma cells (possibly by reducing ROS-mediated loss of ER proteostasis), thereby weakening the functional interaction between dying melanoma cells with antigen-presenting cells (117). Likewise, the heightened autophagic flux harbored by the BRAFV600E inhibitor-resistant melanoma cells, hindered the surface exposure CRT and HSP90, in response to cell death induced by the blockade of the hyperactive MEK pathway (118).

Recently, melanoma cell-associated activation of the oncogenic Wnt/beta-catenin signal was found to prevent antitumor immunity by inhibiting the intratumoural recruitment of dendritic cells and T cells (119). Interestingly, microphthalmia-associated transcription factor (MITF), the crucial melanocytes lineage factor, has been recently shown to misregulate endolysosomal biogenesis and to promote Wnt signaling (120) (as discussed later in more details). Whether the melanoma cell-intrinsic and Wnt-mediated immunosuppressive mechanism described above is regulated by MITF-driven endolysosomal changes has not been investigated yet. However, considering the emerging pro-tumorigenic role of the heightened ELS in melanoma (discussed in further sections), this hypothesis needs urgent validation.

In conclusion, various proteostasis mechanisms leading to the upregulation of cytoplasmic chaperones or expansion of the ELS compartment are recruited by melanoma cells to support cell autonomous adaptation to stress from one hand and to maintain the immunosuppressive microenvironment, from the other hand, ultimately promoting disease progression and drug resistance. However, loss of ER proteostasis along with other housekeeping mechanisms may enable key chaperones like CRT and HSP70, with extracellular immunomodulatory functions, to get exposed on the surface of the dying melanoma cells, and modulate antitumor immunity in response to certain anti-melanoma therapies. These observations highlight the plasticity and context-dependent relevance of proteostasis control in melanoma.

## Degrade and Recycle: Autophagy “On Demand” in Melanoma

Several recent studies have highlighted that the transition from the melanocytes to malignant melanoma cells entails changes in major degradation pathways (121–126). Recent studies have shown that the role of autophagy (Box 2) in carcinogenesis is highly context dependent. On the one hand, it may act as a tumor suppressor by removing damaged and ROS-producing organelles, primarily mitochondria thereby favoring metabolic homeostasis and counteracting metabolic rewiring characteristic of malignant cells (122–124, 127). Moreover, autophagy has been proposed to prevent malignancy by decreasing the risk of genome instability, favoring oncogene-induced senescence, degrading oncogenic proteins, ensuring the maintenance of normal stem cell populations, lowering inflammation, and regulating immune responses (123). On the other hand, in established tumors, autophagy may favor oncogenesis by providing tumor cells with essential amino acids or an alternative energy supply to boost their metabolic need under conditions of nutrient and oxygen deprivation, a typical phenomenon of the tumor environment (66). Moreover, in the tumor microenvironment, cancer cell-associated and stromal cell-associated autophagy engage in a tight cross talk that favors cancer cell dissemination through the blood stream (127, 128), repression of antitumor immunity (127), and exchange of metabolites supporting cancer cell’s metabolic needs (129). Therefore in established tumors, in many instances, albeit not in all, blocking autophagy results in increased therapeutic efficacy [reviewed in Ref. (127)].

Although evidence reporting autophagy alterations during melanoma progression is still incomplete, available knowledge supports a dynamic implementation of autophagy in melanomagenesis and melanoma dissemination. In the early phases of melanoma growth, mRNA and protein expression levels of the pro-autophagic proteins Beclin 1 and LC3 have been found to be lower compared to benign nevi (130). Primary melanoma cells also display reduced expression levels of ATG5, a factor which was associated with a worse prognosis (131). Induction of autophagy by ectopic ATG5 expression or autophagy inducers attenuated clonogenic growth of melanoma cell lines harboring low ATG5 levels (131). Downregulation of ATG5 promoted melanocytes transformation by curtailing oncogene-induced senescence (131). Moreover, the use of the lysosomotropic drug chloroquine (CQ) revealed that primary melanoma cells have a reduced autophagic flux in comparison with melanocytes (132).

Collectively, these studies support the hypothesis that early during melanomagenesis the tumor-suppressive role of autophagy is repressed (132). This phenomenon appears to be regulated by epigenetic mechanisms (133) and, at least in part, by the autophagy-inhibitory activity exerted by the heightened activity of the AKT pathway in primary melanoma (132).

Efficient autophagic capacity is recovered during melanoma progression along with the acquisition of other mutations altering the oncogenic landscape. In line with this, metastatic melanoma cells have a higher autophagic flux in comparison to primary melanoma and melanocytes, which was found to be a crucial transition for their survival and clonogenic expansion (132). This switch toward a restoration in the autophagy capacity associated to the invasive/aggressive melanoma phenotype is further supported by analysis of biopsies from human melanoma patients. Consistently higher LC3B and Beclin 1 expression are found in samples of patients with advanced/metastatic melanoma as compared to non-invasive, primary tumors (134–136). Furthermore, increased levels of autophagy prior to treatment in metastatic melanoma have been shown to predict invasiveness, chemoresistance, and poor patient survival (135).

These data support the concept that autophagy in melanoma is regulated in a somewhat biphasic manner. Early in melanomagenesis autophagic flux is decreased, a condition that may favor accumulation of pro-oncogenic mutations and cellular damage, ultimately supporting malignant transformation. In the later stages of melanoma progression autophagy is reactivated, to serve as a major pro-survival mechanism supporting the high metabolic demands and adaptation to stressful tumor microenvironment conditions (118, 132, 136). In line with this, studies using a melanoma xenograft model showed that compromising melanoma cell-associated autophagy by silencing ATG1 or VPS34 (class III PI3K) expression, increased melanoma cell death induced by leucine deprivation (137). Moreover, in a syngeneic host, murine B16F10 melanoma cells silenced for the expression of ATG5 exhibited a severely reduced growth potential, inability to survive in the blood stream and metastasize (128), thus indicating a general role of pro-autophagic proteins in supporting metastatic melanoma dissemination. Likewise, Beclin 1 silencing in B16F10 melanoma halted tumor growth and increased apoptosis *in vivo* (138) [further reviewed in Ref. (139)].

Interestingly, the expression of the HIF1-responsive gene BCL-2/adenovirus E1B 19 kDa protein-interacting protein 3 (BNIP3), an atypical BH3-only protein contextually implicated in the regulation of both cell death and autophagy (140), is constitutively higher in melanoma cells as compared to normal melanocytes (141). BNIP3 *via* its BH3 domain can displace Beclin 1 from its interaction with Bcl-2, therefore activating autophagy, while through its LIR motif, functions as an autophagy receptor for the clearance of mitochondria (142). In melanoma cells, BNIP3 not only regulates autophagic clearance of ROS-generating mitochondria but is also a key orchestrator of actin-driven formation of PM protrusions (ruffles), melanoma cell morphology, and migration (141). Intriguingly, melanoma cell-associated BNIP3 was also found to regulate the stability of the integrin-associated CD47 a surface molecule acting as a powerful “don’t eat me signal” that favors cancer cell escape from immunosurveillance mechanisms during carcinogenesis, a property that is not shared with ATG5 (141). This suggests that BNIP3 is an essential player in the regulation of melanoma cell proteostasis, a function that is exerted by the dual regulation of the pool of healthy mitochondria, with crucial implications for melanoma bioenergetics and metabolic reprograming, and the composition of the PM, possibly affecting melanoma’s phagocytic barrier. In line with this, co-regulated BNIP3/CD47 mRNA expression is correlated to cancer progression and poor prognosis (141).

In line with a tumor-promoting role of autophagic pathways in advanced melanoma, several studies have shown that pharmacological or genetic inhibition of autophagy increase drug-induced cytotoxicity in melanoma cells (127). With the intensive interest of targeted therapy, great relief came with the initial clinical success of BRAF inhibitors (BRAFi), targeting the V600E mutation in melanoma such as vemurafenib (or PLX4032). Unfortunately, the development of drug resistance and relapse prevailed within 1 year of therapy initiation (143). Although a significant number of studies have highlighted the rationale for the inevitable acquisition of drug resistance in melanoma, the underlying mechanisms still remain largely unsolved (144).

Recently it was reported that patients harboring BRAFi/vemurafenib-resistant melanoma’s displayed heightened levels of autophagy compared with their responsive counterparts, and those patients displaying increased levels of therapy-induced autophagy had drastically lower response rates to BRAFi and poor prognosis (145). Interestingly, the CQ derivative Lys05 potentiated BRAFi-induced cell death and enhanced its antitumor activity in mice bearing MEL624 melanoma xenografts (145), suggesting that targeting the heightened lysosomal pathway displayed by these drug-resistant melanoma cells may be of therapeutic benefit. At the mechanistic level, BRAF inhibition in BRAFV600E-melanoma cells induced ER-stress and the predominant activation of the PERK–eIF2α–ATF4/ATF3 pathway, which in turn promoted cytoprotective autophagy (145). Notably, inhibition of mutant BRAFV600E by vemurafenib increased its interaction with both the ER-associated and cytosolic pools of GRP78, thereby ensuing the ER-stress response (145). Thus, deregulated MAPK signaling in melanoma cells alters chaperone-mediated quality control mechanisms, which in turn triggers protective proteostatic measures, through the cross talk between UPR_ER_ and autophagy. Together, these stress responses alleviate sufficient proteotoxic stress to allow melanoma cells to adapt and evolve molecular mechanisms that circumvent BRAF inhibition and reactivate MEK-based signaling, ultimately leading to increased resistance to proteotoxic stress (145). Altogether, these findings suggest that targeting melanomas’ addiction to autophagy in late stage metastatic disease could compromise vital cellular mechanisms of protection against proteotoxic stress. In line with this, suppression of autophagy has been shown to exacerbate amyloidogenic-stress caused by persistence of aggregate-prone or amyloid-like proteins in other paradigms (146, 147).

However, contrary to several reports indicating a cytoprotective role of autophagy in anti-melanoma therapy, other studies have also reported that the autophagy machinery could favor or promote proteotoxic stress and melanoma killing. For example, an autophagy-dependent, caspase-independent melanoma cell death mediated by ROS generated by the photoactivation of 5-ALA has been shown to dependent on the melanin content, such that only non-pigmented melanoma cells died through autophagy (148). Melanoma killing, both *in vitro* and *in vivo*, in response to the dsRNA mimic, PEI-conjugated polyinosine–polycytidylic acid (pIC), was found to be mediated by the ability of the dsRNA helicase melanoma-differentiation-associated gene 5 (MDA-5) to drive endosome–autophagosome–lysosome fusion events, ultimately leading to NOXA-induced apoptosis (149). These studies suggest that conditions altering the cross talk among proteostatic pathways, or exacerbating fusion events in the endo-lysosomal pathway, may turn off the intrinsic pro-survival function of certain autophagy mediators, thereby amplifying the activation of cell death pathways.

Moreover, it should be also considered that autophagy-independent processes are emerging for autophagy-related genes. Of note, melanin biogenesis [a process known to require some components of the autophagy machinery like WIPI1 (150)] has been recently shown to be negatively regulated by ULK1, independent of its canonical autophagy-inducing role through the ATG13/FIP200 complex and mTORC1 regulation (151). ULK1 depletion increased the levels of melanin and was implicated in MITF-mediated induction of tyrosinase (TYR), the rate-limiting factor in melanin biogenesis (151). Whether ULK1 acquires pro-death properties and mediates ROS-induced cell death in non-pigmented melanoma cells remains to be investigated.

Finally, also the *in vivo* autophagy-independent effects of the first-generation autophagy inhibitors, CQ/hydroxychloroquine (HCQ), should be considered, as recently evidenced in a melanoma mouse model where the key autophagy gene *Atg5* was deleted either in melanoma cells or in the tumor vasculature (128). We found that *in vivo* CQ reduced intratumoural hypoxia and metastasis, while improving chemotherapy response, largely by eliciting tumor vessel normalization. This CQ-mediated effect was independent on endothelial cell-associated ATG5 but involved alterations in the trafficking and degradation mechanisms of the antiangiogenic NOTCH1 receptor, resulting in its activation through the endocytic route (128).

In addition, as mentioned above, autophagosomes can interact/interplay at multiple stages of the endocytic pathway, a vital process that is ruthlessly exploited by melanoma cells to support progression and malignancy (125, 152), thus allowing for additional cross talk between trafficking and degradation mechanisms regulating melanoma proteostasis.

In conlusion, in spite of the emerging evidence indicating that autophagy plays a pivotal role in melanoma, a better understanding of the complex and context-dependent relationship that exists between the (epi)genetic profile of the evolving melanoma cell and its interaction with key microenvironmental factors is required to devise therapeutic strategies aiming at harnessing autophagy in melanoma.

## On the Move: Endo/Lysosomal Signalling in Melanoma

Although aberrations in vesicular trafficking pathways (Box 3) may not be themselves the drivers of tumorigenesis, they may be utilized by cancer cells, on “demand,” to support oncogene-driven proliferation, to facilitate invasion and seeding to other organs, and to increase the plasticity of the interface with other stromal cells (153). Moreover, deranged vesicular trafficking may alter the accurate recycling, delivery, and degradation of proteins, resulting in a new compendium of surface localized proteins and lipids that may alter signaling circuits, adhesion/migratory properties, and invasion mechanisms, favoring cancer progression (153). In line with this, accumulating evidence indicates that vesicle trafficking and lysosomal degradative pathways modulate melanoma plasticity and favor dissemination (125, 127, 154).

Gene expression profiling and experimental evidence indicate that during melanomagenesis, high levels of MITF in melanoma cells are associated with a vesicular trafficking signature hallmarked by high expression of Rab7, TBCD1D16, and Rab27a (155). Moreover, as discussed above, MITF-driven ELS biogenesis results in stimulation of Wnt signaling from the MVBs (120), which in turn support melanoma proliferation. The expression levels of MITF, encoding a basic helix–loop–helix/leucine zipper transcription factor, which is found mutated (156) or amplified in 30–40% of melanoma’s (157), is dynamically altered during melanoma progression depending on the need. In general, a large body of evidence supports the view that increasing levels of MITF expression promote melanoma proliferation and drug resistance (126) [extensively reviewed in Ref. (158)]. However, a recent study also reported that decreased/loss of MITF expression in conjunction with an increased expression/activation of receptor tyrosine kinases (RTKs), like AXL and EGFR in melanoma cells correlated with increased cross-resistance toward MAPK pathway inhibitors and the induction of an invasive melanoma phenotype, suggesting that the oncogenic role of MITF is modulated by its cross talk with RTK pathways (159). Moreover, whether this is linked with MITF-driven alterations in the ELS affecting recycling and signaling properties of melanoma-associated RTKs (160) has not been explored yet.

Microphthalmia-associated transcription factor is a member of the MiT family of transcription factors, which include transcription factor EB (TFEB), the master regulator of lysosome biogenesis and autophagosomes formation, through the regulation of the expression of genes belonging to the coordinated lysosomal expression and regulation (CLEAR) network (161) in an mTOR-dependent manner (162). TFEB has also been reported to be a target of ERK2 and starvation along with ERK2 inhibition promoted TFEB nuclear translocation and consequent stimulation of the autophagic/lysosomal program (161). Although the role for TFEB in melanoma is still understudied, it would be interesting to investigate if blockage of this MAPK–TFEB pathway in combination with leucine starvation is a way to keep autophagy at bay and elicit caspase-dependent apoptotic cell death of melanoma cells (137).

Within the endo-/lysosomal gene cluster identified in melanoma, Rab7 was recently found to be differentially expressed in melanoma cells, compared to normal melanocytes and other non-melanocytic tumor cell lines, and to foster melanoma growth by accelerating migration/invasion dynamics (125). Moreover, promoter-based analysis identified the neural crest lineage master regulator SOX10 and the oncogene MYC as drivers of Rab7 transcription in melanoma (125). Increased expression of Rab7A in human cells, clinical specimens, and mouse models highlighted that this key membrane traffic regulator is an early-induced melanoma driver, to which melanoma become particularly addicted to support their increased invasiveness and metastatic potential (125).

Moreover, a heightened “endo-/lysosomal status” could favor melanoma plasticity, by facilitating the uptake of key nutrients from the extracellular environment required to sustain melanoma cell’s high proliferation rates (163). In line with this, Class I PI3K signaling has been shown to promote macropinocytosis upon oncogene activation in human melanocytes and to recruit Rab7A for their degradation (164). Interestingly, Golgi phosphoprotein 3 (GOLPH3), a Golgi-localizing protein, is encoded by 5p13, a region that is focally amplified in melanoma (165). GOLPH3 is important in endosomal-trans golgi signaling and has been shown to enhance endocytosis in melanoma cells *via* interaction with VPS35. Furthermore, expression of GOLPH3 increased mTOR and AKT activity, whereby in melanocytes GOLPH3 overexpression in conjunction with mutant BRAF or NRAS resulted in increased anchorage-independent and xenograft growth *in vivo* (165).

This heightened endo-lysosomal signature in the early phase of melanomagenesis could be hijacked to plastically support the melanoma phenotype switching paradigm. Melanoma phenotype switching, also referred to as EMT-like transition, is a hallmark of the plastic nature of this cancer. EMT-like transition entails that a reversible expression of a set of genes regulating either a “proliferative signature” or an “invasive signature,” is imposed by key microenvironmental factors (i.e., hypoxia, inflammation) rather than the acquisition and selection of specific pro-metastatic mutations and is reversibly acquired “on demand” to support melanoma escape and proliferation at a new metastatic site. In line with this, melanoma subpopulations have been shown to harbor specific gene expression programs, whereby “proliferative” melanoma cells display MITF^high^, SOX10^high^, and PAX3^high^, in line with the reported function of MITF in driving endosomal biosynthesis and Wnt-mediated proliferation. On the other hand, invasive cells have a MITF^low^, TGF-signaling^high^, ZEB1^high^ signature (166). Moreover, the strength but also the duration of TGF-β signaling, which is hyperactivated in the invasive melanoma cells, is highly affected by the internalization route and the capacity to signal from the endosomes (167). Additionally, E-cadherin a junction protein, present in the proliferative melanoma cells is not only regulated by genetics and epigenetics but also by endocytosis and endosomal recycling. Indeed, EMT-inducing stimuli promote E-cadherin ubiquitination and subsequent lysosomal degradation in a process that requires the sequential activation of Rab5 and Rab7 (168). Altogether, these data support the idea that the dynamics of the endocytic compartment play an important role in the plastic phenotype switching of the melanoma cells.

Taken together, these studies suggest that although the ELS is not a direct driver of melanoma progression, enhanced lysosomal functionality is a key weapon within the melanoma’s arsenal. Moreover, exacerbated ELS may indirectly drive disease progression by accelerating and facilitating invasion or metastasis and by buffering the enhanced proteostatic burden or by supplying additional materials required for melanoma growth, spread, and progression. Finally, its role in shaping the extracellular matrix may also aid in or promote melanoma plasticity.

## Communicate and Educate: Melanoma-Derived Exosomes

Melanoma cells not only rewire intracellular adaptations and degradative pathways to their advantage but also upregulate intercellular communication pathways that enable them to support neighboring melanoma cells and educate the tumor stroma and prime (pre)metastatic sites for subsequent invasion (39, 169). An intercellular messenger system key for melanoma progression and metastasis are the secreted vesicles. Melanoma cells secrete multiple extracellular vesicles, which are highly heterogenic in their origin, composition, and size. The best characterized secreted extracellular vesicles are the exosomes, which are formed in the cell cytoplasm within the multivesicular bodies, trafficked to the cell borders *via* the cytoskeleton and subsequently released upon fusion with the PM. With their small diameters of less than 150 nm, exosomes function as tiny messenger bags, which not only contain proteins but also lipids, DNA, and RNAs. On top of a specific cargo, these messenger bags also contain a specific delivery address, which is indicated by the composition of the membrane, including specific integrin expression patterns, which target them to a specific cell subtype (170). Upon delivery of the exosomes to the target cell, the intracellular signaling can be altered either by direct activation of PM receptors or *via* fusion of the exosome with the PM and subsequent release of its content in the cytosol of the target cell (171). The formation, trafficking, secretion, and uptake of exosomes is regulated at multiple steps by several signaling molecules, including the Rab family of proteins (172, 173).

As mentioned above, melanoma cells have been found to express high levels of key regulators of exosomes formation and trafficking pathways (102), indicating their propensity to recruit the ELS to communicate with their environment. The first functional evidence for a tumor-supportive role for melanoma-derived exosomes was already produced a decade ago when it was shown that the metastatic potential of the aggressive BL6-F10 melanoma cell lines could be transferred to the poorly metastatic BL6-F1 melanoma cell line *via* the exosomes (169). More recently, exosomes secreted by melanoma cell lines have been shown to induce EMT-like transition in normal melanocytes (39). The relevance of exosomes for autocrine/paracrine signaling is also supported by findings showing that exosomes secreted by miRNA22-overexpressing melanoma cells are capable to transfer the miRNA 22-dependent malignant potential of the overexpressing cells to the wild-type cells (40). Besides affecting neighboring melanocytes and melanoma cells, melanoma-secreted exosomes have been reported to have profound paracrine effects on the tumor stroma. One of the tumor stroma components reported to be activated by melanoma-derived exosomes are the endothelial cells of the tumor vasculature. Several reports show the presence of multiple proangiogenic molecules, including IL-6, MMP2, and VEGFA, in melanoma-derived exosomes (41, 45, 102, 174). Another tumor stroma component whose functionality is altered/modulated by the tumor-secreted exosomes are the immune cells. Melanoma-derived exosomes have been shown to prevent monocytes differentiation into dendritic cells and instead skew them toward TGF-β secreting myeloid cells, capable of suppressing T-cell activation and proliferation (175, 176). Moreover MHC class II-containing exosomes isolated from the plasma of melanoma bearing mice have been shown to suppress the tumor antigen-induced immune response in an ovalbumin–antigen model (177). It has been proposed that melanoma-derived exosomes could also induce immune tolerance in the draining lymph nodes by increasing the production of TNF-α by vascular endothelial cells (42).

A unique signaling feature of exosomes is their long half-life in the blood, which makes them excellent long distance messengers for the tumor-induced priming of the (pre)metastatic niche. An elegant study by the group of D. Lyden revealed that melanoma-derived exosomes educate bone marrow-derived cells toward a tumor-promoting phenotype supporting angiogenesis, metastasis, and invasion, by increasing their MET signaling (102). The importance of exosomes in the establishment of the (pre)metastatic niche is also supported by studies showing that melanoma-derived exosomes uptake in the sentinel lymph node increases melanoma cell recruitment, extracellular matrix deposition, and blood vessel activation (45).

Recent patient sample analysis has documented an increase in exosome number and size distribution (44), or higher exosome protein concentrations and a different composition, in patients with advanced disease compared to all other stages (102). In particular, exosomes of melanoma patients were shown to display higher protein levels of melanoma inhibitory activity (MIA), a small soluble protein of 11 kDa secreted by malignant melanoma cells, and S100B, a 21-kDa dimeric calcium-binding protein biomarker for malignant melanoma, known to contribute to melanoma progression at metastatic niche (46, 178). This tight correlation between exosomal protein content and melanoma progression was further confirmed in an *in vitro* study where exosomes from metastatic melanoma cell lines were shown to have a specific protein signature (102, 174).

As mentioned before, certain vital components of the proteostasis system, such as HSP70, are expressed within the endo-lysosomal compartment and at the PM and can be secreted on the membrane of exosomes. A recent study has shown that a peptide aptamer targeting the extracellular domain of HSP70 disrupts the interaction between exosome-associated HSP70 and the toll-like receptor (TLR) 2 on myeloid-derived suppressor cells (MDSCs), potent suppressors of antitumor immunity, thereby lowering their proliferation (47). This effect was particularly evident after cisplatin or 5FU treatment, thus suggesting that blocking the exosomal source of HSP70 may improve the efficacy of anticancer drugs by blunting the immune suppressive functions of melanoma-associated exosomes. This hypothesis still needs to be further proven and evaluated in the context of clinically used anti-melanoma therapies.

Although the true implications of exosome-based communication (both between cancer cells and neighboring cells) is still being unraveled, the studies discussed above highlight a vital role for melanoma-derived exosomes in facilitating and supporting multiple steps of melanoma progression. In that context, melanoma-derived exosomes may hold value as diagnostic and prognostic biomarkers of disease progression.

## Conclusion and Perspectives

Research over the past decades has evidenced the ability of melanoma cells to hijack various homeostatic processes to adapt to the changing and hostile microenvironment and disseminate. One of the most successful strategy adopted by melanoma cells to foster their plasticity is to model regulatory pathways governing proteostasis. This is achieved by the recruitment of a cluster of lysosome- and endolysosome-associated genes, by expanding and modulating the unfolded protein and the HSRs and autophagy/degradative pathways, and by intensifying or even generating novel and robust communication routes within the main nodes of the proteostasis network (schematically illustrated in Figure 1). Perturbing degradation, recycling and trafficking mechanisms alter the functional status of signaling proteins/receptors within the endo-lysosomal network or movement of endocytic cargo to inappropriate destinations (e.g., shifting the cargo from degradation to recycling or secretion). Ultimately, this deranged proteostasis network is reflected in changes of melanoma cell’s proteome and secretome, which plastically fuel melanoma aggressive behavior and shape the microenvironment. However, such a dependency also reveals an intrinsic vulnerability of melanoma cells, which can be exploited therapeutically. For example, autophagy pathways at the late stage of melanoma development, when they become exquisitely important for melanoma survival and growth, can be therapeutically targeted. Understanding how and when sabotage of the altered proteostasis system in melanoma can be harnessed for therapeutic purposes is an outstanding question that will require future studies. Future studies would need to identify the high connectivity points within a network that are more susceptible to lethal perturbations. Research is already progressing in that direction as demonstrated by recent studies that show the increased therapeutic benefit of combining inhibitors of multiple deranged proteostasis pathways. In this context, it would be interesting to model proteostasis network of melanoma cell at the system level, in order to model the deranged nodes and predict lethal perturbations. Finally, these system biology and therapeutic approaches would need to be tested and/or translated *in vivo* to understand the impact of the induced melanoma cell proteotoxicity, at the tumor microenvironmental level.

## Author Contributions

PA conceived the review and largely contributed to the writing of the review. SD, SM, and HM contributed to the writing, checked the correctness of the references, and drafted the figures.

## Conflict of Interest Statement

The authors declare that the research was conducted in the absence of any commercial or financial relationships that could be construed as a potential conflict of interest.

## Abbreviations

5-ALA, 5-aminolevulinic acid; AKT, protein kinase B; ATG, autophagy-related protein; ATG6, activating transcription factor 6; Beclin 1, BH3-only domain protein; BNIP3, BCL-2/adenovirus E1B 19 kDa protein-interacting protein 3; BRAFi, BRAF inhibitors; CHIP, co-chaperone carboxyl terminus of heat-shock cognate 70 (HSC70)-interacting protein; CLEAR, coordinated lysosomal expression and regulation; CMA, chaperone-mediated autophagy; CQ, chloroquine; CRT, calreticulin; CTLA, cytotoxic T-lymphocyte antigen-4; DAMPs, damage-associated molecular patterns; eIF2α, PERK-eukaryotic translation initiation factor 2 subunit 1; ELS, endo-lysosomal system; EMT, epithelial-to-mesenchymal-like transition; ER, endoplasmic reticulum; ERAD, ER-associated degradation; ESCRT, endosomal sorting complex required for transport; GOLPH3, Golgi phosphoprotein 3; GPC, general proteostasis control; GRM1, metabotropic glutamate receptor 1; GRP78, 78 kDa glucose-regulated protein; HCQ, hydroxychloroquine; HSC70, heat-shock cognate 70; HSF1, heat shock transcription factor 1; HSR, heat shock response; ICD, immunogenic cell death; IL, interleukin; IRE1, inositol-requiring element 1; LAMP1, lysosomal-associated membrane protein 1; LC3B, microtubule-associated proteins 1A/1B light chain 3B; LEs, late endosomes; LIR, LC3-interacting region; MAPK, mitogen-activated protein kinase; MAPS, misfolded-associated protein secretion; MDA-5, melanoma-differentiation-associated gene 5; MDSCs, myeloid-derived suppressor cells; Mel, melphalan; MITF, microphthalmia-associated transcription factor; mTOR, mechanistic (formerly “mammalian”) target of rapamycin; MVBs, multivesicular bodies; MYC, V-myc avian myelocytomatosis viral oncogene homolog; NBR1, neighbor of BRCA1 gene 1; NFκB, nuclear factor kappa-light-chain-enhancer of activated B cells; NO, nitric oxide; p62, nucleoporin p62; PD-1, programed cell death-1 protein; PERK, ER membrane the (PKR)-like ER kinase; PI3K, phosphatidylinositol-4,5-bisphosphate 3-kinase; PI3P, phosphatidylinositol-3-phosphate; pIC, PEI-conjugated polyinosine–polycytidylic acid; Rab7, Ras-related protein Rab7; ROS, reactive oxygen species; RTKs, receptor tyrosine kinases; SNARE, soluble NSF attachment protein (SNAP) receptor; SOX10, sex-determining region Y (SRY)-box 10 (SOX10); TFEB, transcription factor EB; TGF, transforming growth factor; TLR, toll-like receptor; TYR, tyrosinase; UPR, unfolded protein response; UPS, ubiquitin–proteasomal system; VEGF, vascular endothelial growth factor.
